# Genomics of COVID-19: molecular mechanisms going from susceptibility to severity of the disease

**DOI:** 10.1186/s40246-020-00273-5

**Published:** 2020-06-10

**Authors:** Ruty Mehrian-Shai, Giuseppe Novelli, Vasilis Vasiliou, Jessica Watt, Juergen K. V. Reichardt

**Affiliations:** 1 Pediatric Hemato-Oncology, Sheba Medical Center, Tel Hashomer, Israel; 2 Department of Biomedicine and Prevention, University Tor Vergata, Rome, Italy; 3 Department of Environmental Health Sciences, School of Public Health, Yale University, New Haven, USA; 4 College of Public Health, Medical and Veterinary Sciences, James Cook University, Smithfield, QLD Australia; 5 Australia Institute of Tropical Health and Medicine, James Cook University, Smithfield, QLD Australia

The current COVID-19 pandemic (https://www.who.int/dg/speeches/detail/who-director-general-s-opening-remarks-at-the-media-briefing-on-covid-19%2D%2D-11-march-2020) has highlighted the importance of science and medicine, specifically public health, in our modern societies. Countries have taken different approaches to the pandemic.

Science and medicine will play an important role in our way forward. Specifically, genetics and genomics will be central in discovering variations in virus strains and their impact on patients’ susceptibility, progression, and outcome. Additionally, the human and zoonotic hosts’ ability to fend off the virus and the severity of disease in patients will have genomic elements. Furthermore, the question of long-term immunity to COVID-19 will likely have a genomic basis which should be investigated. Some of these genetic and genomic investigations will undoubtedly be suitable for publication in *Human Genomics*. We, therefore, expressly welcome submissions of manuscripts on such subjects to the thematic series on the “Genomics of COVID-19: molecular mechanisms going from susceptibility to severity of the disease.”

As citizen-scientists, we also have a responsibility toward society as citizens first and foremost but also specifically as scientists working on improving the lives of all humans. Political aspects of the COVID-19 pandemic and its origins can and should not be ignored as the needs of all humans: rich and poor, various ethnicities, rural vs. urban, different health and political systems, etc. These aspects, if fitting within the scope of the journal, will also be considered for publication in this thematic series in *Human Genomics*.

In summary, the COVID-19 pandemic will impact all of humanity. Basic and medical research will have an important role to play in the way forward. Genomics will make important contributions to our understanding of origins of and susceptibility to the disease, its progression, outcome, possible reinfection, and much more. We look forward to a lively forum in the thematic series on the “Genomics of COVID-19: molecular mechanisms going from susceptibility to severity of the disease” of this journal, *Human Genomics*, to publish and share novel and important insights into the pandemic spanning all aspects falling within the scope of the journal at this vital time.

## Abbreviation

COVID-19: Corona virus disease 2019

